# Detection of Novel duck reovirus (NDRV) using visual reverse transcription loop-mediated isothermal amplification (RT-LAMP)

**DOI:** 10.1038/s41598-018-32473-4

**Published:** 2018-09-19

**Authors:** Zhili Li, Yuejia Cai, Guozhi Liang, Saeed El-Ashram, Minmin Mei, Wenjing Huang, Xiaowen Li, Wenfeng Li, Cheng He, Shujian Huang

**Affiliations:** 1 College of Life Science and Engineering, Foshan University, 18 Jiangwan Street, Foshan, 528231 Guangdong Province China; 2 Faculty of Science, Kafrelsheikh University, Kafr El-Sheikh, Egypt

## Abstract

Here we present a visual reverse transcription loop-mediated isothermal amplification (RT-LAMP) assay for detecting the gene encoding the σB major outer-capsid protein of novel duck reovirus (NDRV). A set of primers, composed of two outer primers, two inner primers and two loop primers, was designed based on the gene of interest. The LAMP reaction was conducted in a traditional laboratory water bath at 65 °C for 50 min. We compared the performance of calcein/Mn^2+^ and SYBR Green I dyes, as well as electrophoresis on agarose gel stained with GoldView nucleic acid dye to detect the RT-LAMP-amplified products and all assays could be employed to discriminate between positive and negative specimens in visible or UV light. Our data showed that there is no cross-reaction with other viruses and the RT-LAMP technique displayed high sensitivity for detecting NDRV with a minimal detection limit of 200 fg RNA input. This assay was more sensitive than conventional PCR in detecting NDRV both in natural and experimental infection. In conclusion, the RT-LAMP technique was remarkably sensitive, specific, rapid, simple and profitable for the identification of NDRV.

## Introduction

A novel duck reovirus (NDRV) disease, called “spleen necrosis disease,” “new liver disease in Muscovy ducks” or “duck hemorrhagic-necrotic hepatitis,” was recently found among several duckling species, including shelducks, Pekins, wild mallards and Muscovy in China^1^. Similarly, avian reovirus (ARV) infection was recorded in Muscovy ducks (*Cairina moschata*) in south western Poland during the summer 2012^2^. NDRV is a member of the genus *Orthoreovirus* in the family Reoviridae^3^.

The disease can be distinguished from Muscovy duck reovirus (MDRV) infections by clinical presentation of hemorrhagic and necrotic lesions in the liver and spleen and 5–50% mortality rates. Furthermore, the existing commercial vaccines against ARVs or MDRVs fail to prevent NDRV infection and transmission. A reverse transcription loop mediated isothermal amplification (RT-LAMP) provides a rapid and precise detection assay for viral pathogens with high sensitivity and specificity^4^. This technique is profitable and convenient, only requires a constant temperature water bath and averts some deficiencies, including high necessity for equipment, tremendous cost, lengthened examination period and low sensitivity^5^. In comparison with the equipment required for traditional polymerase chain reaction (PCR) and quantitative polymerase chain reaction (qPCR) assays, RT-LAMP is easy to conduct in resource-limited laboratory settings in underdeveloped countries^6^. RT-LAMP has been widely exploited in clinical diagnosis of various viral pathogens including porcine epidemic diarrhea virus (PEDV), transmissible gastroenteritis virus (TGEV), classic swine fever virus (CSFV), H10N8 subtype of influenza A virus, porcine deltacoronavirus (PDCoV), Zika virus (ZIKV), chikungunya virus (CHIKV), Rift Valley fever virus (RVFV), St. Louis encephalitis virus (SLEV), yellow fever virus (YFV), dengue virus serotypes 1–4, Japanese encephalitis virus (DENV1–4) and West Nile virus (WNV)^7–10^. The ARV structural and molecular compositions are generally similar to those of mammalian reovirus (MRV). However, some of the ARV biological properties differ from mammalian reovirus, which have been shown elsewhere^11,12^. The S3 segment encoding the σB protein of duck and MRVs is structurally similar to ARV σB gene^13^. Reverse transcription PCR (RT-PCR) on both sigma C (σC) and sigma B (σB)-encoding genes followed by restriction fragment length polymorphism (RFLP) analyses were employed to characterize Tunisian ARV isolates^14^. Furthermore, the highly variable sequences of the S3 and M3 have been reported to differentiate between novel duck reovirus (NDR), MDRV and avian reovirus^15^. Thus, a simple, rapid and sensitive diagnostic technique for detection of NDRV is required. The potential application of RT-LAMP assay using the S3 gene of NDRV-NPO3 strain for specific diagnosis of NDRV infection with limited sensitivity and without its utilization in the detection of NDRV in naturally- and experimentally-infected ducks have been reported^16^. In this study, we developed and evaluated a RT-LAMP assay targeting the gene encoding the σB major outer-capsid protein to detect NDRV in naturally suspected NDRV-infected ducks and experimentally infected ducklings with NDRV.

## Materials and Methods

### Ethics statement

This study was approved by the Animal Ethics Committee of the College of life science and engineering, Foshan University, Guangdong, China. The College did not issue a number or ID to this animal study, because the studied ducks are not an endangered or protected species. Specimen collection was carried out based upon the protocol issued by the Animal Ethics Committee of the College of life science and engineering. Furthermore, all methods were performed in accordance with the relevant guidelines and regulations.

### Virus isolates for specificity evaluation

Novel duck reovirus disease SH12 (NDRV- SH12) (preserved in the laboratory of Preventive Veterinary Medicine), was isolated from the infected ducks in Guandong province, China, Muscovy Duck Reovirus (MDRV-S12), Avian Reovirus (ARV-S1133), Duck Disease Virus (DPV), Duck Hepatitis Virus (DHAV), Duck Newcastle Disease Virus (NDV), H9 subtype avian influenza virus (AIV), H5 subtype AIV and duck Tanzuru virus (DTMUV) were stocked at −80 °C in our laboratory at College of life science and engineering, Foshan University, China. A total of 15 ducks were collected from the Guangdong region suspected to be infected with NDRV. The specimens were sent to the College of life science and engineering, Foshan University, China and examined upon arrival.

### Experimental infection of ducklings

Ten 1-day-old Muscovy ducklings were randomly divided into 2 groups (5 birds per group). Group 1 ducklings were intraperitoneally injected with 0.2 mL (10^4.00^ELD_50_) of the NDRV allantoic fluid and ducklings in group 2 were intraperitoneally injected with physiological saline and served as the control group. All ducklings were observed hourly for 72 h post-infection (hpi).

### RNA extraction

Total RNA was extracted employing an EasyPure Viral DNA/RNA kit (Transgen, Beijing, China) according to the manufacturer’s protocol and was stored immediately at −80 °C until use.

### Reverse transcriptase PCR (RT-PCR)

RT-PCR was performed employing a PrimeScript One Step RT-PCR Kit (TaKaRa, Japan). Primers S-F (5′-GCTTTTTGAGTCCTCAGCGTG-3′) and S-R (5′-GATGAATAGGCGAGTCCCGC-3′) were used to amplify the corresponding S3 segment encoding sigma B gene. For RT-PCR, 2.5 µL of extracted viral RNA was mixed with a reaction mixture containing 1 µL primer (10 pmol), 1 µL Prime Script One Step Enzyme mix, 8 µL RNase-free H2O and 12.5 µL 2 × One Step Buffer. Reverse transcription was performed at 50 °C for 30 min. The cycling conditions for the PCR were 94 °C for 2 min, followed by 32 cycles of denaturation (94 °C for 10 s), annealing (58 °C for 30 s) and extension (72 °C for 1 min), followed by a final extension at 72 °C for 7 min.

### Cloning and sequencing

The amplified PCR products were subjected to agarose gel electrophoresis, excised from the gel and purified using an Agarose Gel DNA Purification Kit (TaKaRa, Japan). The PCR products were cloned into the pMD19-T vector according to the manufacturer’s instructions (TaKaRa, Japan). After the recombinant plasmid was transformed into DH5α competent cells, the plasmid DNA purified using the E.Z.N.A.® Plasmid Mini Kit I (Omega Bio-Tek, USA) and quantified by spectrophotometric analysis. Three clones were sent to Shanghai Sangon Bioengineering Ltd. to be sequenced using the aforementioned primer pair and the sequence was analyzed.

### Design of primers for LAMP

**Figure 1:**
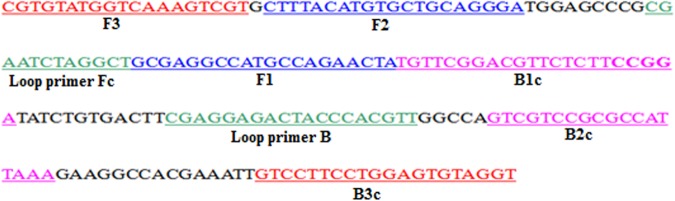
Location of the NDRV RT-LAMP primer in the gene encoding the σB major outer-capsid protein.

Based on the gene encoding the σB major outer-capsid protein (GenBank accession number JQ866923.1; 1104 bp) of NDRV (DRB-QY strain), a set of six primers consisted of two outer primers (F3 and B3), two inner primers (FIP and BIP) and two loop primer (Loop primer Fc and Loop primer B) was generated employing the Primer Explorer V4 software (http://primerexplorer.jp/e/). The forward inner primer (FIP) composed of the complementary sequence of F1c and F2. The backward inner primer (BIP) consisted of B1c and the complementary sequence of B2c (B2). The outer primers consisted of the forward outer primer F3 and the backward outer primer B3 (the complementary sequence of B3c). In addition, there are two loop primers (Loop primer Fc and Loop primer B) to accelerate the amplification reaction as previously described^17^. The positions of the LAMP primers used in this study are shown in Fig. 1 and Table 1. Primers were synthesized by Shanghai Sangon Bioengineering Ltd, China.

**Table 1:** LAMP primers for NDVR detection.

Primer name	Primer sequence (5′-3′)	Primer position (bp)	Primer length (bp)
F3	CGTGGATGGTCAAAGTCGT	901–919	19
B3	ACCTACACTCCAGGAAGGAC	1 080–1 099	20
FIP	TAGTTCTGGCATGGCCTCGC-CTTTACATGTGCTGCAGGGA	921–940 969–988	40
BIP	TGTTCGGACGTTCTCTTCCGGA-TTTAATGGCGCGGACGAC	1 047–1 064 989–1 010	40
Loop primer F	AGCCTAGATTCGCACTCCG	950–968	19
Loop primer B	CGAGGAGACTACCCACGTT	1 023–1 041	19

### RT-LAMP assay optimization

The RT-LAMP reaction was carried out in a final volume of 25 μL. To optimize the RT-LAMP assay, the parameters, including the amplification temperature (59–66 °C) and the amplification time (10–60 min) were determined. The parameters of the reaction mixtures, such as the ratio of inner and outer primers (2:1–12:1), the ratio of loop and outer primers (0:0.2–6:1) and the ratio of calcein and manganese ion (1:8–1:128) was described independently. In addition, the concentrations of betaine (0–1.5 mM; Sigma, USA), dNTPs (0.2–0.7 mM; Promega, USA), MgSO4 (0–6 mM; Sigma, USA), AMV reverse transcriptase (0.06–0.16 U/µL; Takara, Japan), Bst DNA polymerase (0.8–0.48 U/µL; New England Biolabs, USA) and 10 × ThermoPol buffer (0–4 µL) were sequentially optimized.

### Detection of RT-LAMP products

The RT-LAMP reactions for NDRV were carried out at 65 °C for 50 min, followed by denaturation for 5 min at 85 °C to cease the reaction. The RT-LAMP-amplified products were visualized on 2% agarose gel stained with GoldViewTM under UV transillumination. The calcein visualization RT-LAMP assay can be inspected under UV light, which emitted strong green fluorescence. Moreover, products were visualized directly by addition of 1.0 µL of 1:10 diluted SYBR Green I (Invitrogen, USA). To determine the sequence specificity, the cleavage site of RT-LAMP amplified fragment was analyzed by SeqBuilder software and the single Hpa II restriction site was screened. Two microliters of RT-LAMP amplified products were digested with restriction enzyme HhaI (New England Biolabs, USA) at 37 °C for 2 h according to the manufacturer’s protocol and the restriction enzyme digestion products were analyzed on 2% agarose gel electrophoresis. Each reaction was repeated at least three times.

### Specificity of RT- LAMP identification

To determine the specificity of RT-LAMP assay, RT-LAMP was conducted with the different nucleic acid templates extracted from Muscovy duck reovirus (MDRV-S12), avian reovirus (ARV-S1133), duck plague virus (DPV-YF15), duck hepatitis virus (DHAV-3), duck Newcastle disease virus (NDV-SS14), H9 subtype avian influenza virus (NH/2013), H5 subtype avian influenza virus (GM/2014) and duck virus (DTMUV-GD13) under the aforementioned conditions. Each virus was investigated at least three times.

### Sensitivity of detection amongst RT-LAMP visualized by calcein, conventional RT-LAMP and RT-LAMP visualized by SYBR Green I

Detection sensitivities between RT-LAMP visualized by calcein, RT-LAMP analyzed by agarose gel and RT-LAMP visualized by SYBR Green I were compared using 10-fold serial dilutions of the virus-positive total RNA extracts (NDRV) and recombinant plasmid. The resulting concentrations were as follows: 2 ng, 200 pg, 20 pg, 2 pg, 200 fg, 20 fg and 2 fg. Detection limits were determined by the lowest input nucleic acid concentration at which a positive result was apparent.

## Results

### Optimization of RT-LAMP assay for NDRV detection

All of the possible variables were investigated to enhance the amplification efficiency. Three replications were carried out for each trial. RT-LAMP was performed under different reaction temperatures from 59 °C to 65 °C. The results showed that RT-LAMP at 65 °C produced very clear and bright bands. Therefore, 65 °C was selected as the optimal temperature of NDRV RT-LAMP reaction. The reaction time indicated that 50 min was adequate for reaction completion. The ratio of calcein and manganese ion (1:8–1:128) was analyzed and the color was obvious at the ratio of 1:12. Consequently, the ratio of 1:12 was selected as the optimal ratio to use in proceeding experiments. The effect of Mg^2+^ ion concentration was analyzed. Magnesium sulfate (Mg^2+^) was added at a concentrations ranging from 0 to 6 mM reaction mixtures containing positive sample. Upon gel analysis, bands were obvious at 3 mM Mg^2+^. When the concentration was greater than 3.0 mM (lane 4~7), the reaction efficiency decreased but tended to be stable. Therefore, 3.0 mM was chosen as the best reaction concentration for NDRV RT-LAMP. Moderately various yields were detected with the different amounts of betaine employed and a concentration of 0.5 mM was found to be proper for a more distinct pattern. The size of the ladder increased as the concentration of dNTPs increased and a concentration greater 0.3 mM was found to be sufficient. AMV reverse transcriptase concentration of 0.14 U/µL was found to be appropriate for the amplification efficiency of NDRV RT-LAMP reaction. The reaction efficiency decreased as the concentration of the AMV enzyme concentration increased. However, a more intense band was observed with 2.5 µL of 10 × Buffer, a 10:1 ratio of inner vs. outer primers (FIP + BIP 2 M, F3 + B3 0.2 µM) and a ratio of 4:1 ring and outer primers (LoopF + LoopB 0.8 M, F3 + B3 0.2 µM) and 0.32 U/L of Bst DNA polymerase (Figs S1; 2–3).

**Figure 2:**
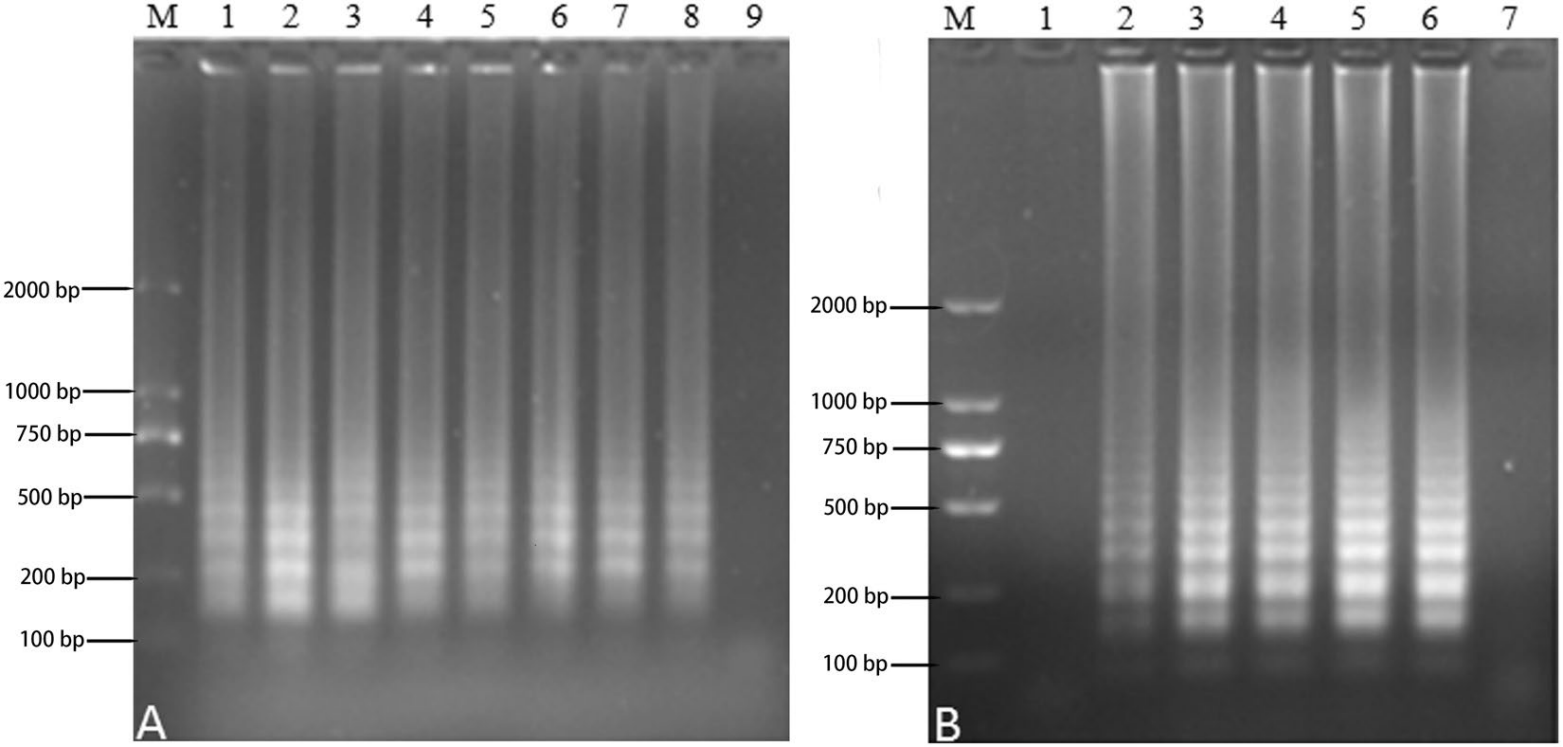
Procedures optimization of the NDRV RT-LAMP reaction. (**A**) The effect of temperature: M, 2000 bp DNA Marker; lanes 1–8 (66 °C, 65 °C, 64 °C, 63 °C, 62 °C, 61 °C, 60 °C and 59 °C, respectively) and lane 9: ddH_2_O (negative control; NC). (**B**) The effect of time: lanes 1–6 (10, 20, 30, 40, 50 and 60 min, respectively); lane 7 NC.

**Figure 3:**
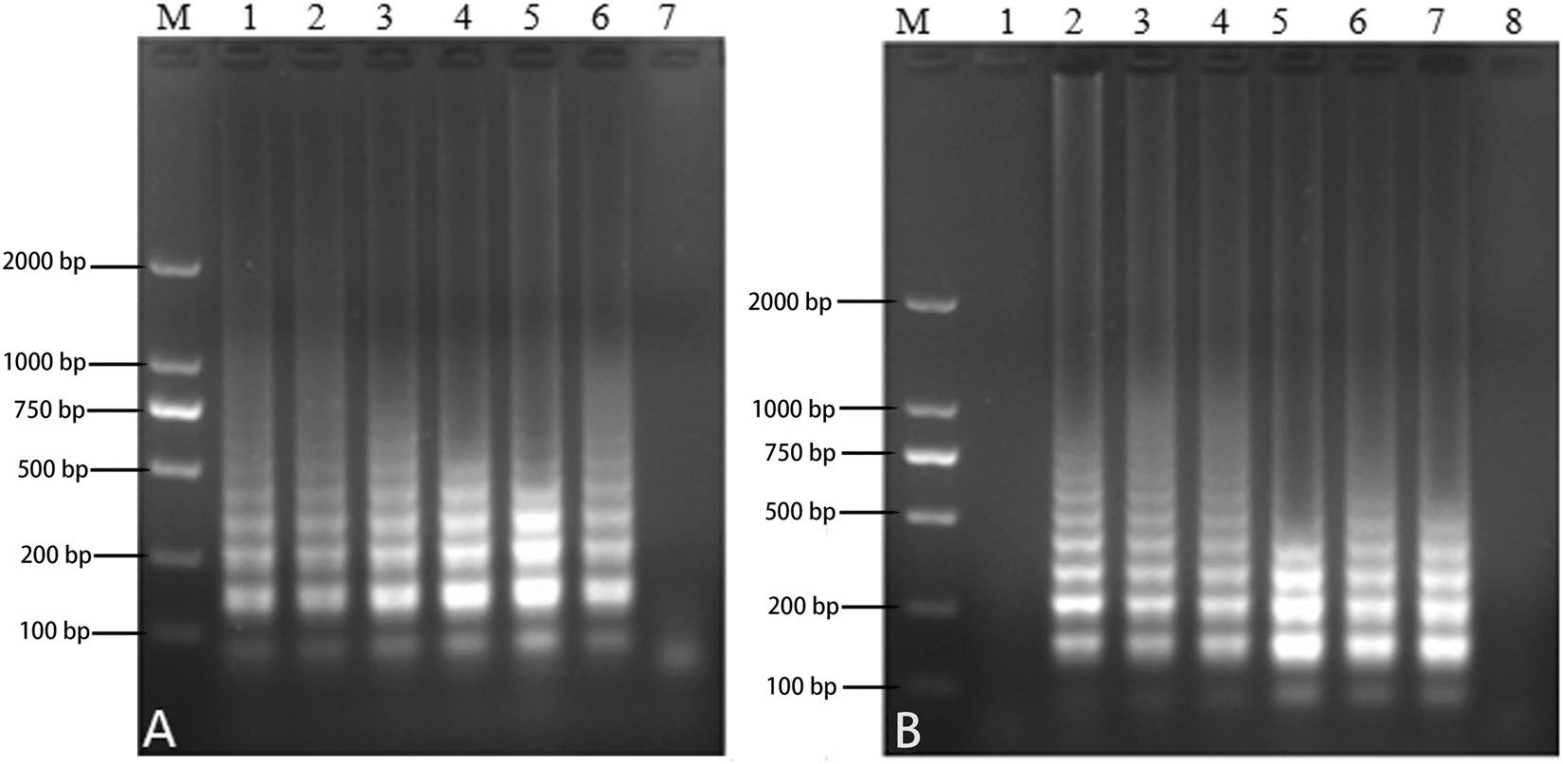
Primer optimization of the NDRV RT-LAMP reaction. (**A**) The effect of the ratio of inner and outer primers: M, 2000 bp DNA Marker, lanes 1–6 (2:1, 4:1, 6:1, 8:1, 10:1 and 12:1, respectively) and lane 7 NC. (**B**) The effect of the ratio of loop (Loop primer Fc and Loop primer B) and outer primers (F3 and B3): M, 2000 bp DNA Marker, lanes 1–7 (0:0.2, 1:1, 2:1, 3:1, 4:1, 5:1 and 6:1, respectively); lane 8 NC.

Taken together, the optimized parameters of the RT-LAMP reaction system were as follows: 3 mM MgSO_4_, 0.5 mM betaine, 0.3 mM dNTPs, 0.14 U/μL AMV reverse transcriptase, 0.32 U/μL Bst DNA polymerase large fragment, 2.5 μL10 × ThermoPol buffer, the ratio of inner (2.0 μM; FIP and BIP) and outer (0.2 μM; F3 and B3) primers and the ratio of loop (0.8 μM; Loop primer Fc and Loop primer B) and outer primers (0.2 μM; F3 and B3) with an incubation condition at 65 °C for 50 min in a water bath. The RT-LAMP-amplified products were resolved on 2.0% agarose gel and checked by visual investigation employing calcein under UV or the production of insoluble manganese/ magnesium phosphate and by electrophoresis on GoldView^TM^ nucleic acid stained agarose gels under UV light. Moreover, the RT-LAMP products were also visually detected by adding SYBR Green I dye.

### Analysis of RT-LAMP digestion products

**Figure 4:**
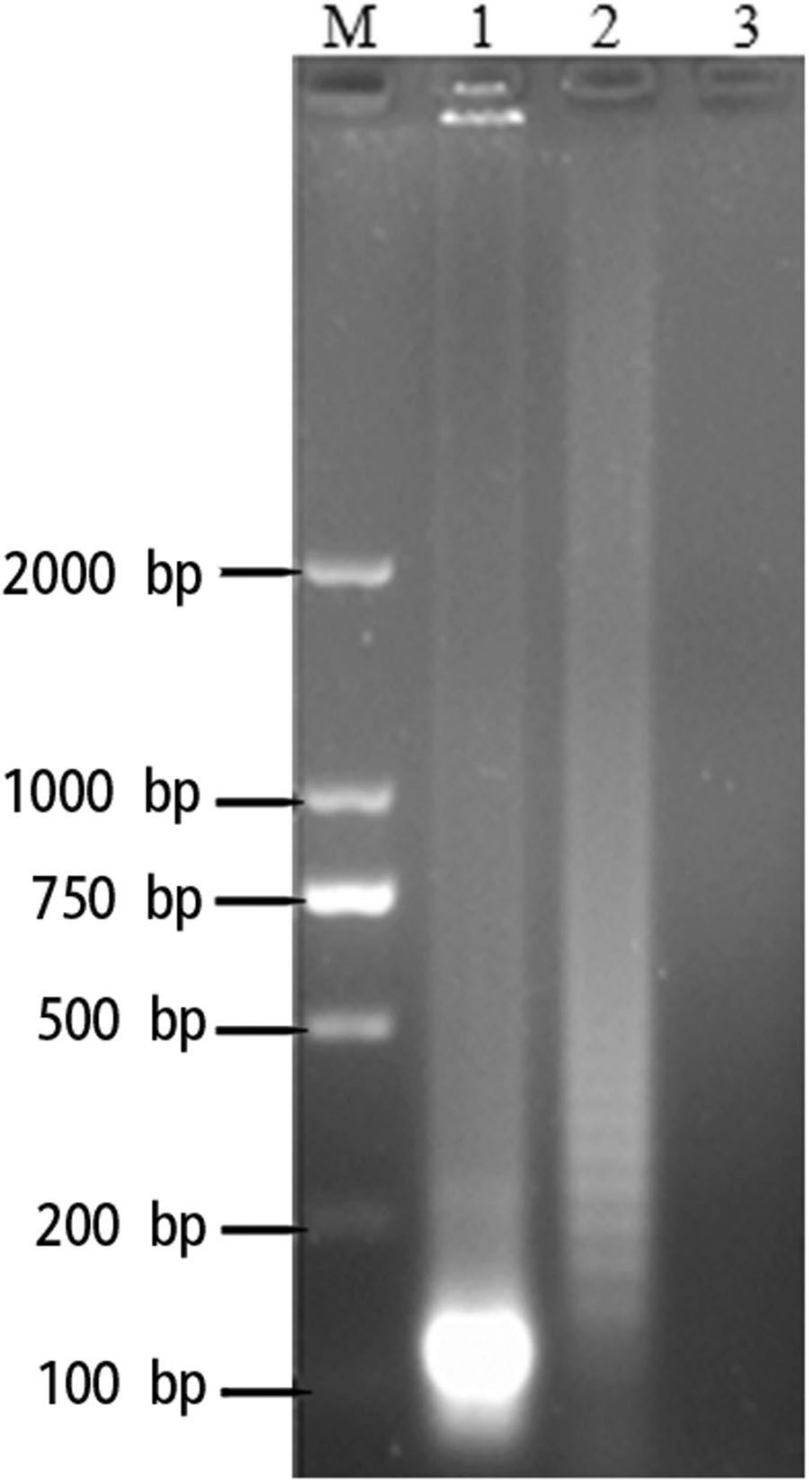
Agarose gel electrophoresis results for NDRV RT-LAMP reaction products and their HhaI restriction enzyme digestion results. M, 2000 bp DNA Marker; lane 1(HhaI restriction enzyme digestion results); lane 2 ladder-like pattern bands; lane 3 NC.

Electrophoresis of the RT-LAMP products showed that all the positive LAMP reactions produced characteristic ladder-like pattern bands on agarose gel. HhaI restriction enzyme digestion and electrophoresis gave a strong band at approximately 100 bp corresponding to the predicted sizes (a mixture of 127 bp and 114 bp) (Fig. 4).

### Specificity of RT-LAMP identification

**Figure 5:**
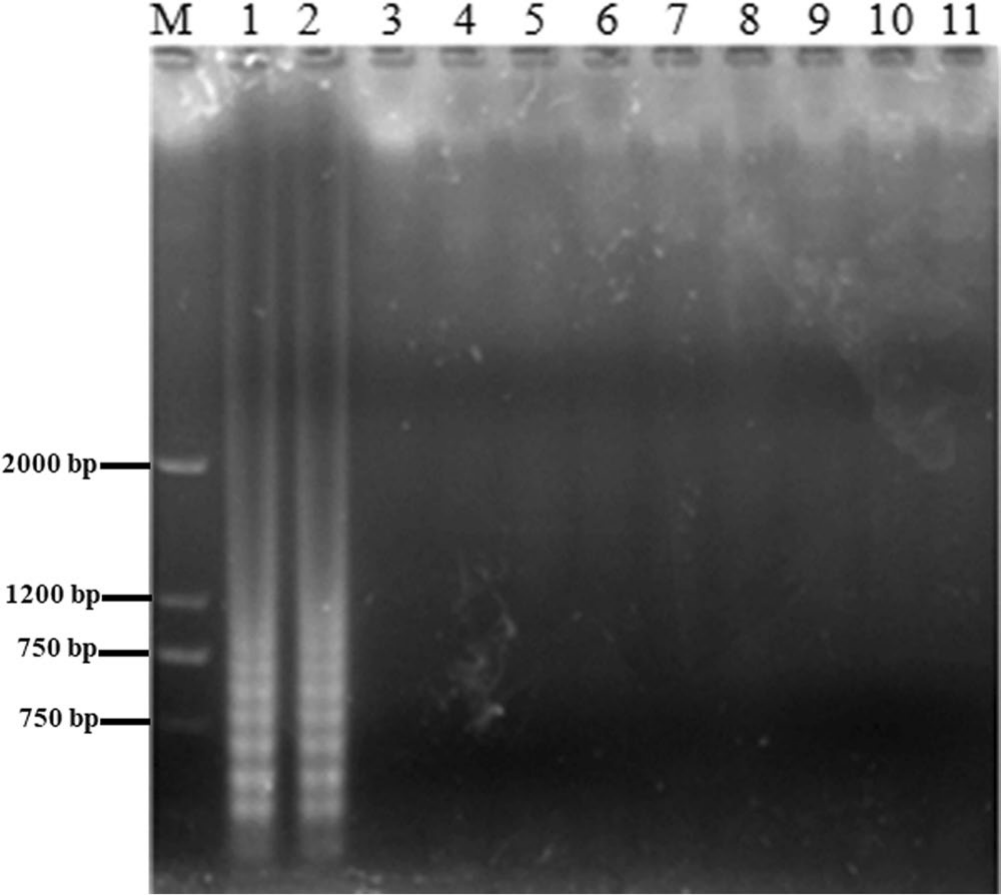
Identification specificity of the NDRV RT-LAMP assay. Samples were resolved on 2.0% agarose gels. LAMP was carried out with the different sources of nucleic acids. Lane M, 2000 bp DNA ladder; Lane 1, recombinant plasmid; Lane 2 NDRV sample; Lanes 3–11, MDRV, ARV, DPV, DHAV, NDV, H9 AIV, H5 AIV, DTMUV and NC.

As illustrated in Fig. 5, only when the recombinant plasmid and RNA from NDRV were present. The RT-LAMP reaction was positive and the RT-LAMP-amplified products were detected, showing a typical ladder-like pattern on gel electrophoresis, which indicated that stem-loop DNA with inverted repeats was generated, whereas RT-LAMP reaction was negative for double distilled water (negative control; NC) and the other viruses tested, indicating that the LAMP reaction was highly specific to NDRV.

### RT-LAMP sensitivity comparison

The sensitivity of RT-LAMP assay visualized by calcein was first judged by the dilution of RNA input and recombinant plasmid into 2 ng, 200 pg, 20 pg, 2 pg, 200 fg, 20 fg and 2 fg, respectively and the result of this assay was compared with those of RT-LAMP visualized by SYBR Green I and RT-PCR analyzed by agarose gel (Fig. S2A–F). The comparison study revealed that the detection limit of RT-LAMP visualized by calcein was 200 fg. This result is in accord with that of RT-LAMP visualized by SYBR Green I and RT-LAMP analyzed by agarose gel, but it is more sensitive than of conventional RT-PCR (20 pg detection limit).

### RT-LAMP assay evaluation with field samples (suspected NDRV infection) from Guangdong province

A total of 15 clinical samples were used with their tissue mixed together for each individual duck and 12 clinical sample organs (including heart, liver, spleen, lung, thymus and bursa) were used separately from two NDRV-infected ducks in this study. The detailed results of detection (specimen by specimen) are listed in Tables 2 and 3. The comparative evaluation of RT-LAMP and conventional RT-PCR revealed that RT-LAMP and RT-PCR were consistent when the commonly affected organs were mixed together (Table 2). The viral RNAs of NDRV were detected in 20% (3/15) of the 15 duck studied. However, the positive rates of conventional RT-PCR and RT-LAMP for separately affected organs NDRV with were 83% (10/12) and 92% (11/12), respectively as indicated in Table 3.

**Table 2:** Detection results of NDRV in suspected clinical specimens by conventional RT-PCR and RT-LAMP (commonly affected organs, heart, liver, spleen, lung, thymus and bursa mixed together for each duck).

Sample no	Origin in Guandong province	Duck varieties	Conventional RT-PCR assay	Calcein visualization RT-LAMP assay
1	Si hui	Muscovy duck	−	−
2	Jiang men	Muscovy duck	−	−
3	Dan zao	Muscovy	−	−
4	Ding hu	Muscovy	−	−
5	Zhao qing	Muscovy	−	−
6	He shun	Muscovy duck	−	−
7	Tai shan	Hybrid Muscovy Duck	−	−
8	Kai ping	Hybrid Muscovy Duck	−	−
9	Qing yuan	Hybrid Muscovy Duck	−	−
10	Dan zao	Hybrid Muscovy Duck	−	−
11	San shui	Muscovy duck	+	+
12	Xin xing	Hybrid Muscovy Duck	+	+
13	Si hui	Hybrid Muscovy Duck	+	+
14	Xin xing	Hybrid Muscovy Duck	−	−
15	Ding hu	Hybrid Muscovy Duck	−	−

**Table 3:** Detection results of NDRV in clinical samples from NDRV-naturally infected ducks by conventional RT-PCR and RT-LAMP (commonly affected organs evaluated separately).

sample	Conventional RT-PCR assay	Calcein visualization RT-LAMP assay
**Duck no A**		
Heart	+	+
Liver	−	−
Spleen	+	+
Lung	+	+
Thymus	+	+
Bursa	+	+
**Duck no B**		
Heart	−	+
Liver	+	+
Spleen	+	+
Lung	+	+
Thymus	+	+
Bursa	+	+

### RT-LAMP assay evaluation with experimentally infected ducklings

**Figure 6:**
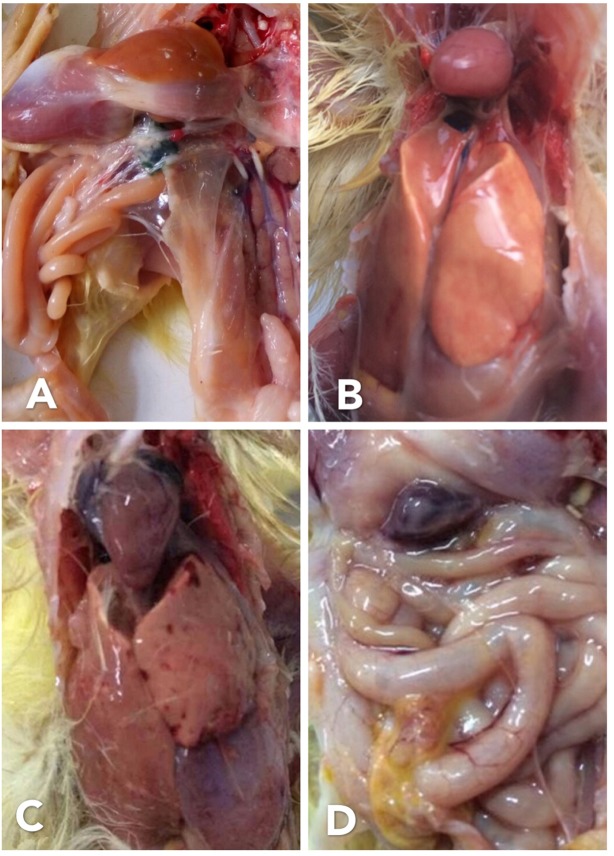
Macroscopic lesions of NDRV affected 1-day-old ducklings. (**A**) liver and spleen in physiological saline-injected ducklings; (**B**) hepatomegaly and pleural exudates with yellow discoloration 48 hpi; (**C**) hepatomegaly with brittle texture and patchy hemorrhagic necrosis 72 hpi; (**D**) red darken splenomegaly, enlarged heart and inflated intestine 72 hpi.

A total of ten 1-day-old ducklings were randomly allocated into two groups (five ducklings each). Group 1 animals were received intraperitoneal inoculation of NDRV allantoic fluid (Table 4). Ducklings from group 2 were intraperitoneally inoculated with physiological saline and served as the control group. Ducklings were followed hourly for 72 hours with their corresponding uninfected-control ones. All ducklings in the infected group died with 72 hours post-infection (hpi). The gross anatomical lesions of the ducklings showed an enlarged liver (hepatomegaly) and pleural exudates with yellow discoloration 48 hpi and hepatomegaly with brittle texture, plaque bleeding and necrosis, red darken splenomegaly, patchy hemorrhagic necrosis, bursal necrosis, renal bleeding, enlarged heart and inflated intestine 72 hpi (Fig. 6).

**Table 4:** Detection results of NDRV in experimentally infected ducklings by conventional RT-PCR and RT-LAMP (commonly affected organs evaluated separately).

No	Sample (Death time)	Conventional RT-PCR assay	Calcein visualization RT-LAMP assay
1	Liver (24 hpi)	−	−
2	Stool (24 hpi)	−	−
3	Serum (24 hpi)	−	−
4	Heart (48 hpi)	−	−
5	Liver (48 hpi)	+	+
6	Lung (48 hpi)	+	+
7	Brain (48 hpi)	−	+
8	Heart (48 hpi)	−	−
9	Liver (48 hpi)	−	−
10	Lung (48 hpi)	+	+
11	Brain (48 hpi)	+	+
12	Stool (48 hpi)	−	+
13	Stool (48 hpi)	−	+
14	Serum (48 hpi)	+	+
15	Serum (48 hpi)	+	+
16	Serum (72 hpi)	+	+
17	Heart (72 hpi)	−	−
18	Liver (72 hpi)	+	+
19	Spleen (72 hpi)	+	+
20	Brain (72 hpi)	+	+
21	Stool (72 hpi)	+	+
22	Stool (72 hpi)	+	+
23	Heart (72 hpi)	+	+
24	Liver (72 hpi)	+	+
25	Spleen (72 hpi)	+	+
26	Lung (72 hpi)	+	+
27	Brain (72 hpi)	+	+
28	Serum (72 hpi)	+	+

Twenty eight samples were collected at 24, 48 and 72 h, respectively from different affected organs, including heart, liver, spleen, lung and brain as well as anal swab (sticky stool) and serum were collected. Among the 21 NDRV LAMP-positive specimens, 18 were positive RT-PCR (Table 4). The RT-LAMP rapid detection assay was 10.7% (3/28) higher than that of the traditional RT-PCR method. Thus, the conventional RT-PCR method had low sensitivity and is inappropriate for use in diagnosis of NDRV.

## Discussion

NDRV was isolated and identified from various duck species, for example, Muscovy, Mule, Peking and Sheldrake ducks^18^. The infected ducks revealed the fundamental clinical symptoms with hemorrhage and necrosis in the liver results in high mortality rates in ducks. In China, NDRV causes tremendous economic losses to the duck industry and most of the affected ducks, particularly ducklings died within 72 hpi. Therefore, it is crucially required to develop an easy, rapid, highly sensitive and specific diagnostic method for NDRV detection. In this study, we employed RT-LAMP to identify NDVR as a serious pathogen for duck industry in Guangdong province, China. Specificity of the RT-LAMP primers was investigated exploiting Muscovy Duck Reovirus (MDRV), Avian Reovirus (ARV), Duck Disease Virus (DPV), Duck Hepatitis Virus (DHAV), Duck Newcastle Disease Virus (NDV), H9 subtype avian influenza virus (AIV), H5 subtype AIV and duck Tanzuru virus (DTMUV). The utilization of the six specific primers that recognize distinct regions on the gene encoding the σB major outer-capsid protein of NDRV assured high specificity of the template nucleic acid amplification. Our data showed that there was no cross-reaction between NDVR and other examined viruses. The RT-LAMP specificity detection of NDRV was in accord with other studies about specificity of RT-LAMP reaction in viral detection^7–10,19^. The RT-LAMP assay in the detection of NDRV displayed sufficient sensitivity in addition to its higher specificity.

When the nucleic acid and plasmid quantities were checked by the RT-LAMP visualized by calcein assay, the detection limit was found to be 200 fg RNA, which was similar to that of RT-LAMP analyzed by agarose gel and RT-LAMP visualized by SYBR Green I. However, the detection limit of the conventional PCR (20 pg RNA). These results are in line with those of previous studies^20–24^. Interestingly, the current assay is more sensitive than the previously reported one^16^. Furthermore, the sensitivity of RT- LAMP in NDRV-infected ducklings (known) and field samples of ducks (suspected NDVR infection) was found to be higher than that of conventional PCR assay. Although the current study is based on a small number of experimental animals, RT-LAMP is a simple and powerful amplification method for the rapid diagnosis and early detection of experimentally NDVR- infected ducklings within 48 hpi.

The greater sensitivity is due to the high amplification efficiency of the RT- LAMP assay. Moreover, there is no time loss for thermal alteration under isothermal conditions in RT-LAMP. Others have reported that the enzymes used in LAMP are more resistant to inhibitory components in clinical samples^25–29^. Furthermore, it has been reported that PCR assay is more prone to PCR inhibitors in samples, which interfere with the amplification and sensitivity of PCR in comparison with LAMP assay^30,31^. We compared the performance of both calcein and SYBR Green I and our results indicated that both assays could be utilized to differentiate between positive and negative samples in visible or UV light.

The RT-LAMP-amplified products were detected by visual inspection employing calcein and SYBR Green I dyes, as well as by electrophoresis on agarose gels. As a result of the high amplification efficiency of LAMP^31,32^ and binding affinity of SYBR Green I to DNA^33^, the sensitivity of LAMP detection using calcein (the fluorescent emissions from calcein and the production of insoluble manganese/magnesium phosphate)^34^ and SYBR Green I was very high. The visual inspection result with calcein and SYBR Green I dyes was found to match with gel electrophoresis. Thereby, the visual inspection of LAMP amplified products by employing calcein and SYBR Green I dyes instead of gel electrophoresis made the RT-LAMP assay more rapid and simple.

This one-step RT-LAMP established in this study will provide an effective assay for the rapid diagnosis, surveillance and the examination of molecular epidemiology of NDRV for both developed and underdeveloped countries.

## Electronic supplementary material

Supplementary information
