# Supplementary material for: Detection of Novel duck reovirus (NDRV) using visual reverse transcription loop-mediated isothermal amplification (RT-LAMP)
Source: Sci Rep. 2018 Sep 19;8:14039. doi: 10.1038/s41598-018-32473-4 (PMC6145877; doi:10.1038/s41598-018-32473-4)
Supplement: Supplementary file 1 — Supplementary information [file 41598_2018_32473_MOESM1_ESM.pdf]

# Supplementary Materials

## Detection of Novel duck reovirus (NDRV) using visual reverse transcription loop-mediated isothermal amplification (RT-LAMP)

¶Zhili Li[1a], ¶Yuejia Cai[1a], Guozhi Liang[a], Saeed El-Ashram[a,b,*], Minmin Mei[a], Wenjing Huang[a], Xiaowen Li[a], Wenfeng Li[a], Cheng He[a], ShujianHuang[a,*]

[a]College of life science and Engineering, Foshan university, 18 Jiangwan street, Foshan 528231, Guangdong province，China.

[b]Faculty of Science, Kafrelsheikh University, Egypt.

¶Zhili Li and Yuejia Cai contributed equally to this work

*Correspondence to:

Saeed El-Ashram (saeed_elashram@yahoo.com);

Shujian Huang (huangshujian@fosu.edu.cn)

**Supplementary 1A-F Figure**. Reagent optimization for the NDRV RT-LAMP reaction. (A) The effect of $MgSO_4$: M, 2000 bp DNA Marker; lanes 1-7 (0, 1, 2, 3, 4, 5, and 6 mM, respectively); lane 8 NC. (B) The effect of betaine: M, 2000 bp DNA Marker; lanes 1-4 (0, 0.5, 1, and 1.5 mM, respectively); lane 5 NC. (C) The effect of dNTPs: M, 2000 bp DNA Marker; lanes 1-6 (0.1, 0.2, 0.3, 0.4, 0.5, and 0.6 mM, respectively); lane 7 NC. (D) The effect of AMV reverse transcriptase: M, 2000 bp DNA Marker; lanes 1–6 (0.06, 0.08, 0.1, 0.12, 0.14, and 0.16 U/μL, respectively); lane 7 NC. (E) The effect of Bst DNA polymerase: M (2000 bp) DNA Marker; lanes 1-6 (0.08, 0.16, 0.24, 0.32, 0.4, and 0.48 U/μL, respectively); lane 7 NC. (F) The effect of 10x ThermoPol buffer: M, 2000 bp DNA Marker; lanes 1-6 (0, 1, 2, 2.5, 3, and 4 μL, respectively); lane 7 NC.

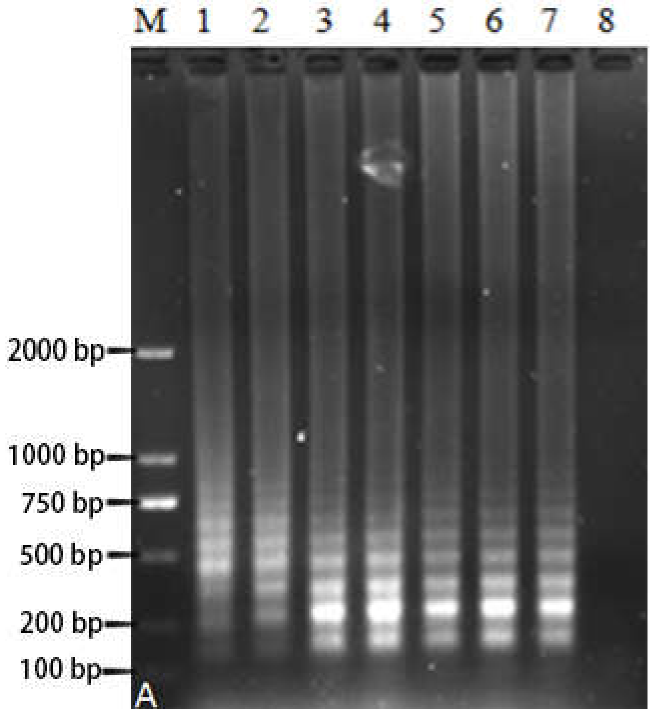

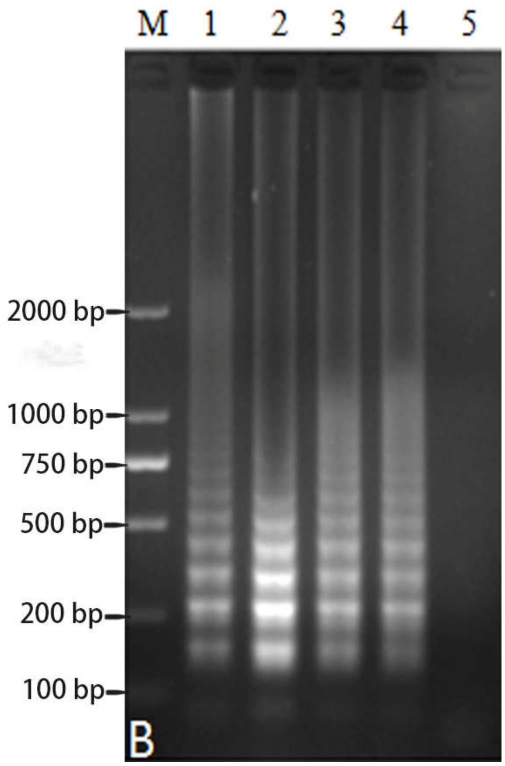
M 1 2 3 4 5
2000 bp
1000 bp
750 bp
500 bp
200 bp
100 bp
B

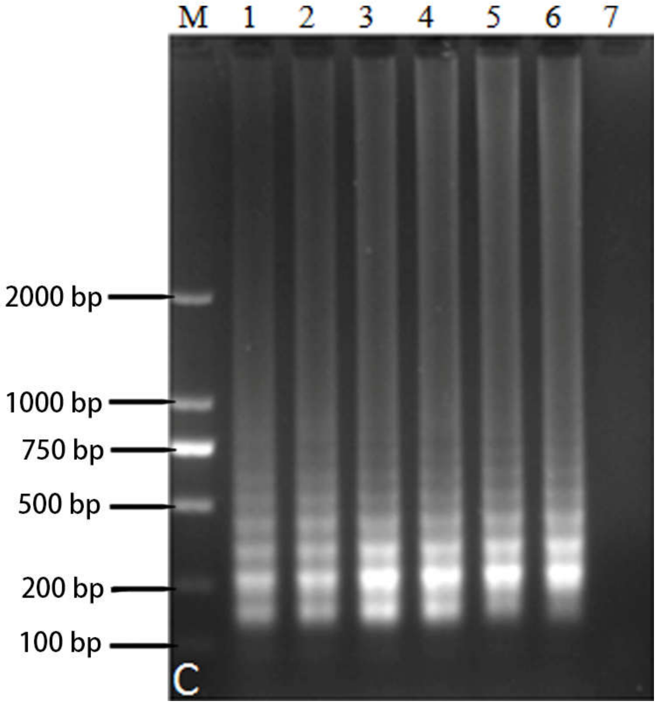
M 1 2 3 4 5 6 7
2000 bp
1000 bp
750 bp
500 bp
200 bp
100 bp
C

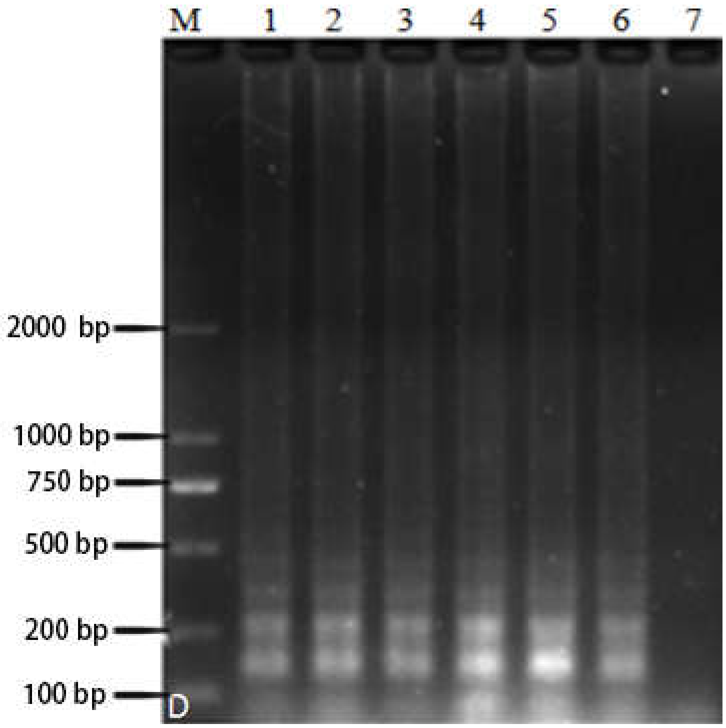
M 1 2 3 4 5 6 7
2000 bp
1000 bp
750 bp
500 bp
200 bp
100 bp
D

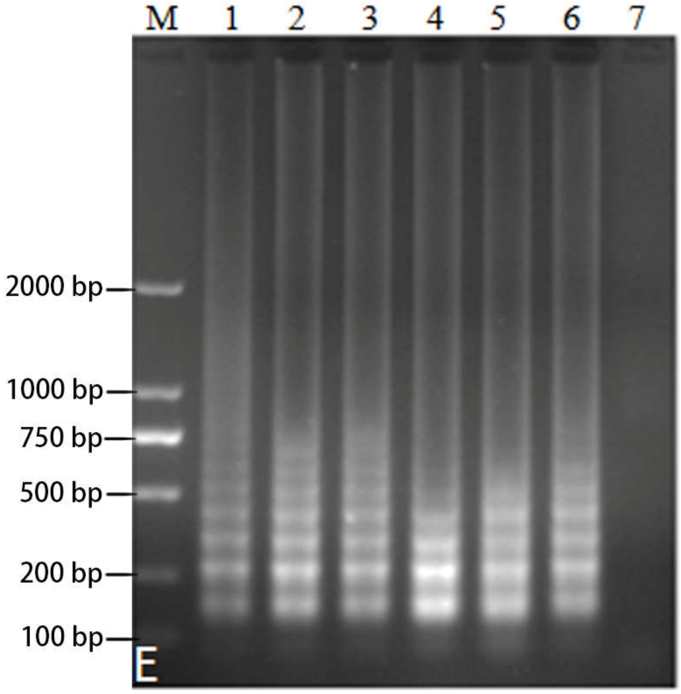
M 1 2 3 4 5 6 7
2000 bp
1000 bp
750 bp
500 bp
200 bp
100 bp
E

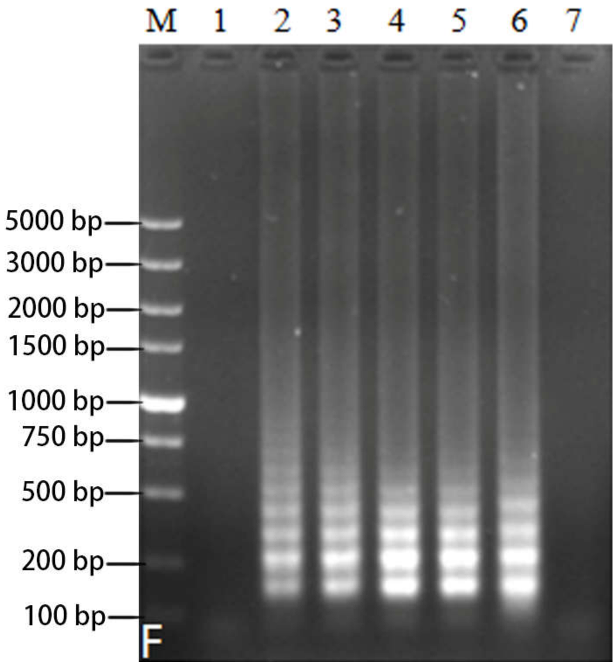


**Supplementary 2A-F Figure.** Detection limit comparison between different amplification assays. Detection of NDRV and recombinant plasmid by calcein (using 2 ng, 200 pg, 20 pg, 2 pg, 200 fg, 20 fg, and 2 fg of input target RNA (A) and recombinant plasmid (B). Gel electrophoresis of RT-LAMP products for NDRV (C) and recombinant plasmid (D) at the indicated concentrations. Tube and lane 8 used as negative controls. (E) RT-LAMP assay visualized by SYBR Green I and conventional RT-PCR (GoldView$^{TM}$) of NDRV at various concentrations. Lane M indicates a 2000 bp DNA ladder, and no amplification was seen for the negative control. (F) Conventional RT-PCR sensitivity (Lane M: DNA marker: 2000 bp DNA marker, 1: 2 ng; 2: 200 pg; 3: 20 pg; 4: 2 pg; 5: 200 pg; 6: 20 fg; 7: 2 fg; 8: ddH$_2$O).

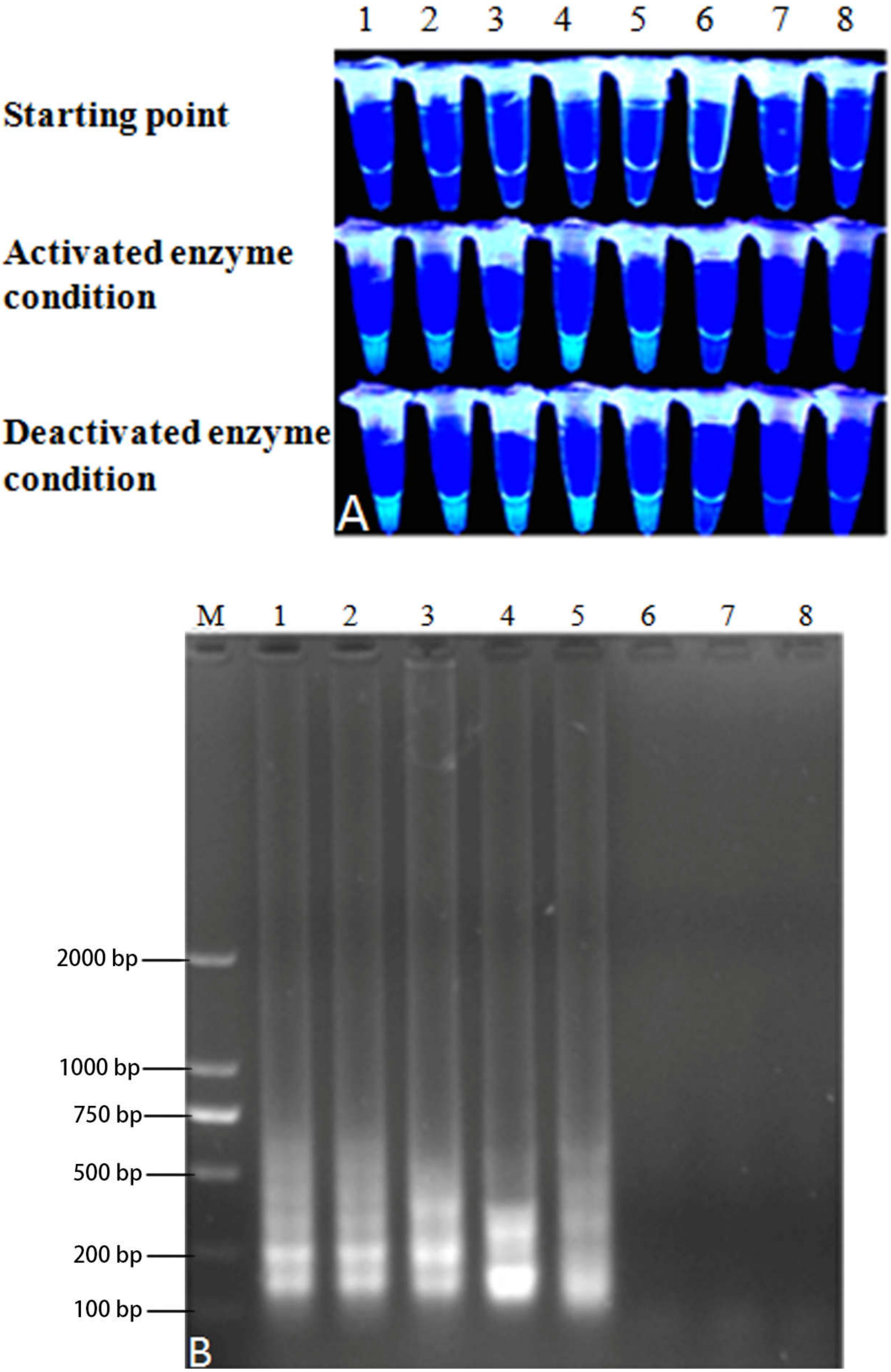

1 2 3 4 5 6 7 8
Starting point
Activated enzyme condition
Deactivated enzyme condition
A
M 1 2 3 4 5 6 7 8
2000 bp
1000 bp
750 bp
500 bp
200 bp
100 bp
B

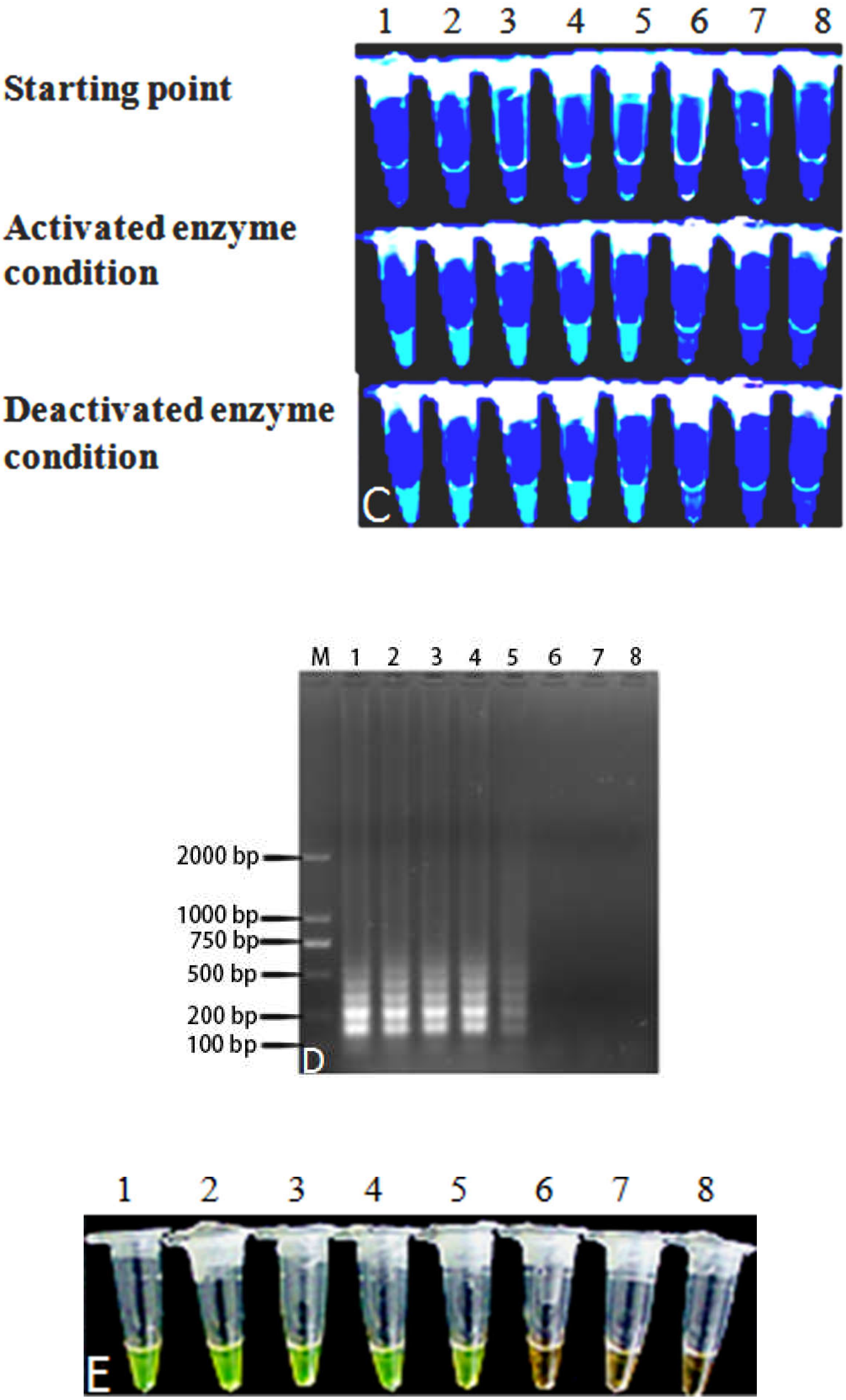
1 2 3 4 5 6 7 8
Starting point
Activated enzyme condition
Deactivated enzyme condition
C
M 1 2 3 4 5 6 7 8
2000 bp
1000 bp
750 bp
500 bp
200 bp
100 bp
D
1 2 3 4 5 6 7 8
E

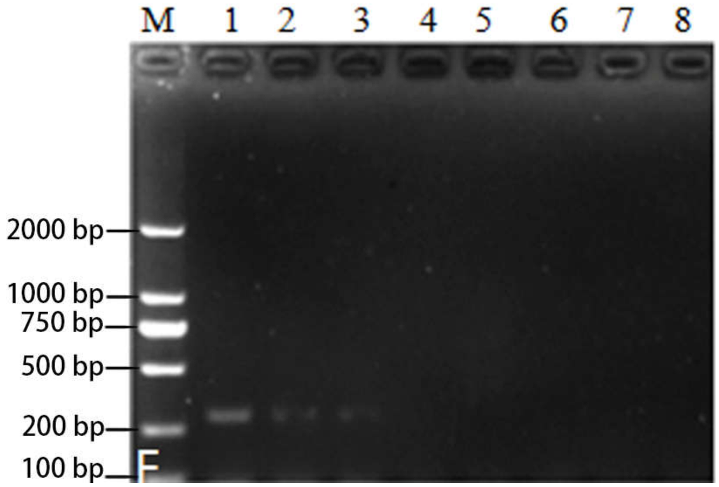
M
1
2
3
4
5
6
7
8
2000 bp
1000 bp
750 bp
500 bp
200 bp
100 bp
